# Cold Exposure and Health Effects Among Frozen Food Processing Workers in Eastern Thailand

**DOI:** 10.1016/j.shaw.2014.10.004

**Published:** 2014-10-18

**Authors:** Anamai Thetkathuek, Tanongsak Yingratanasuk, Wanlop Jaidee, Wiwat Ekburanawat

**Affiliations:** 1 Department of Industrial Hygiene and Safety, Faculty of Public Health, Burapha University, Chonburi, Thailand; 2 Department of Public Health Foundations, Faculty of Public Health, Burapha University, Chonburi, Thailand; 3 Occupational Medicine Center, Samitivej Sriracha Hospital, Chonburi, Thailand

**Keywords:** cold, illness, health surveillance

## Abstract

Frozen food processing workers work under a cold environment which can cause several adverse health effects.This study explored factors affecting workers' health in the frozen food industry in Thailand. Participants comprised 497 workers exposed to a cold working environment and 255 office workers who served as the controls.

Data were collected by a survey on the work environment, and the interview of workers for abnormal symptoms. The exposed group had the following characteristics: 52.7% male, overall average age of 27 (SD 6.6) years old, attained elementary education (Grade 4 and Grade 6) (54.1%), married (48.9%), smokers (21.3%), alcohol consumption (31.0%), duration of work was between 1 and 5 years (65.2%), working 6 days a week (82.7%), 1–5 hours of overtime per week (33.8%), office workers (33.9%); work category: sizing (6.9%), peeling (28.3%) dissecting (22.2%), and in the warehouse (8.6%). The temperature in the work environment ranged from 17.2°C to 19.2°C in most sections, −18.0°C in the warehouse, and 25°C in the office areas. Warehouse workers had more abnormal symptoms than controls including repeated pain in the musculoskeletal system (OR 11.9; 95% CI 6.12–23.45), disturbance throughout the body (OR 4.60; 95% CI 2.00–10.56), respiratory symptoms (OR 9.73; 95% CI 3.53–26.80), episodic finger symptoms (OR 13.51; 95% CI 5.17–35.33).

The study results suggest that workers' health should be monitored especially with regard to back and muscle pain, respiratory symptoms, episodic finger symptoms, and cardiovascular symptoms. Health promotion campaigns such as antismoking and reduction of alcohol consumption should be established because smoking and alcohol consumption are contributing factors to the pathogenesis of Raynaud's phenomenon and peripheral vascular disorders such as hypertension and heart disease.

## 1 Introduction

As one of the world's main food production hubs, Thailand is famous for its frozen food industry. The seafood industry inevitably needs labor to work in many different sections like shrimp beheading, peeling, sizing, dissecting, and so on.

Frozen food processing workers have been exposed to potential health hazards including physical, biological, chemical, and psychosocial work environments [1,2]. Low temperatures are necessary in the production of industrial frozen food, which keep the maintains the quality of fresh food for longer. However, it can be dangerous causing the body core temperature to drop. Accompanied with wind speed and humidity levels, low temperature can affect workers' health [1,3–7].

Although there has been no report on work-related cold stress in Thailand [8], there are several studies that examine the effects of a low temperature working environment and its impact on heath in other locations [3–9]; therefore we should pay attention to these impacts because there are a large number of warehouse workers whose jobs are located in low temperature work environments [9]. After exposure to low temperature, symptoms may not appear immediately. This delay period might distract health care personnel from considering low temperature as the cause of adverse health effects [3,4,9].

Low temperature working environments can cause various diseases [3–7,9] if there is no proper policy in place to control the adverse health effects from cold exposure. Cold exposure may affect various organs such as the respiratory system, musculoskeletal system (usually at temperature below 10 degrees [2]), and cause skin disorders such as rash and hives (urticaria) [11], and cold-associated trauma such as Raynaud's phenomenon [12], frostbite, trench foot, chilblains, and hypothermia.

It is evident that cold work environments can cause adverse health effects [1,4–7,9,10,13]; however, in Thailand, studies on cold exposure and health effects are limited. This study aims to explore the health effects of working in cold environments, to determine factors causing abnormal symptoms in frozen food industrial workers, and to provide basic information to monitor health risks resulting from cold exposure.

## 2 Materials and methods

This is a cross-sectional study in which data were collected from April to September 2013.

### 2.1 Study population and participants

The study population comprised workers exposed to cold work environments who worked in two frozen food factories in Rayong Province, Thailand. The study participants were calculated using the formula for simple logistic regression analysis [14], where *n* was the sample size required, *P* was the rate of the event based on Lekcharoen et al [15] who found that the proportion of workers who were exposed to cold frequently for more than 3 hours a day was 61.4% (*p* = 0.614) and *P*_1_ − *P*_2_ is the difference of the event between physical hazard exposed and nonexposed groups in which the minimum difference was 0.15.

Substituting the values in the formula thus defined the error (α) of 5% (=1.96) and the statistical power (1 − β) of 90% (=1.28). The calculated sample size was 442.7 ≅ 443. Because this study explored many variables, therefore, the sample size [14] when *n*_p_ was the adjusted sample size, and *n*_1_ was the calculated sample size was made using the formula for simple logistic regression analysis. *R*^2^ was the coefficient of multiple logistic regression, and for this study was set at 50% (*R*^2^ = 0.50). The calculated sample size using the formula was 886 individuals.

All participants were permitted to decline or withdraw at any time from the study without penalty. Those who agreed to participate signed an informed consent form. The Institutional Review Board of Burapha University provided ethical approval for the study protocol.

### 2.2 Tools and data collection

#### 2.2.1 Interview

Participants were recruited to the study based on voluntary basis and informed consent was obtained. The interview schedule consisted of five parts: Part 1—Sociodemographic characteristics such as sex, age, education, marital status, smoking history, and drinking history. Part 2—Current working history, number of working hours per day, number of working days per week, time to relax outside of work per day. Part 3—Health effects; cold exposure symptoms such as *repeated pain in the musculoskeletal system* (back pain and muscular pain), *symptoms throughout the body* (discomfort, shivering, itching after cold exposure, entire body cold), *respiratory symptoms* (asthma, respiratory wheezing, cough, excessive sputum, runny nose), *episodic finger symptoms* (darkening of fingers, reddening of fingers, finger pain, toe pain), *face and skin symptoms* (urticarial, face pain), *peripheral circulation symptoms* (blurry vision, headache, confusion), *cardiovascular system* (pallor of fingers, chest pain, arrhythmia). The symptoms were rated by a score of two levels (0–1); where 0 = no symptoms and 1 = has symptom. The interview schedule was verified by two occupational medicine physicians, and an occupational health specialist, then underwent a trial before use.

#### 2.2.2 Working environment data

Secondary data of workplace temperature monitoring were used in this study. A real-time digital thermometer was used to monitor workplace temperature.

### 2.3 Data analysis

A statistical analysis package (IBM Corp. Released 2012. IBM SPSS Statistics for Windows, Version 21.0. Armonk, NY: IBM Corp.) was used for data analysis. Sociodemographic characteristics, work history, and health effects were described in terms of percentages, means and standard deviations. Factors affecting health were analyzed using logistic regression–backward elimination (*p*-remove = 0.10) to determine the relationships between age, sex, smoking, drinking, duration of work (years) and seven types of abnormal symptoms: (1) repeated pain in the musculoskeletal system, (2) symptoms throughout the body, (3) respiratory symptoms, (4) episodic finger symptoms, (5) face and skin symptoms, (6) peripheral circulation symptoms, (7) cardiovascular system symptoms.

## 3 Results

### 3.1 Demographic characteristics

Although 886 was calculated as the sample size for this study, there were 752 (85%) participants which consisted of 497 individuals exposed to cold and 255 controls who worked in offices. Among the exposed group, 52.7% were male, 62.0% were 21–30 years old, 54.1% attained elementary education, 48.9% were married, 21.3% were smokers with a mean smoking duration of 8.45 (SD 6.63) years, 31.4% was drinkers (Table 1).

### 3.2 Current work history

Duration of work among the study group ranged from 0.08 to 22 years, with an average of 2.23 (2.70) years, working 8 hours a day or more. The majority (82.7%) worked 6 days per week. The average amount of overtime was 3.48 hours per week (Table 2).

The temperature in the work environment of the study subjects ranged from 17.2°C to 19.2°C in most sections, and −18.0°C in the warehouse. Workers in sizing, peeling, dissecting, and warehouse sections were exposed to cold hazards from the work environment, process water, and processing products. The temperature in the office areas was 25°C.

### 3.3 Health effects resulting from cold exposure

The participants reported that they had abnormal symptoms, which included musculoskeletal system symptoms, discomfort, respiratory symptoms, episodic finger symptoms, face and skin symptoms, peripheral circulation symptoms, and cardiovascular symptoms (Table 3).

### 3.4 Factors affecting health effects

Multiple logistic regression analysis revealed that factors affecting repeated pain in the musculoskeletal system were sex and work section. Women working at sizing, peeling, dissecting, and in the warehouse sections has a higher risk of having back and muscle pain with the odds of 1.816 (95% CI: 1.186–2.781), 5.966 (95% CI: 3.045–11.691), 1.433 (95% CI: 0.866–2.371), 3.436 (95% CI: 2.097–5.629), and 11.962 (95% CI: 6.123–23.445), respectively.

Factors affecting symptoms throughout the body were sex and work section. Men working in the warehouse were at higher risk of having symptoms throughout the body with the odds of 1.794 (95% CI: 1.219–2.641), and 4.597 (95% CI: 2.002–10.556), respectively.

Factors affecting respiratory symptoms were gender, smoking, and section. Female, smokers, and working in the warehouse were at higher risk of having respiratory symptoms with the odds of 1.888 (95% CI: 1.227–2.905), 1.607 (95% CI: 0.924–2.793), and 9.731 (95% CI: 3.534–26.797), respectively.

Factors affecting episodic finger symptoms were sex and work section. Women working in the sizing and warehouse sections were at higher risk of having hand and finger disorders with the odds of 1.645 (95% CI: 1.119–2.419), 2.479 (95% CI: 1.113–5.438), and 13.514 (95% CI:5.169–35.327), respectively.

Factors affecting face and skin symptoms were sex, age, and section. Being female, older workers, and working in the warehouse section resulted in a higher risk of having face and skin symptoms with the odds of 1.932 (95% CI: 0.936–3.987), 3.509 (95% CI: 1.323-9.308) and 7.858 (95% CI: 3.171–19.471), respectively.

Factors affecting peripheral circulation symptoms were sex and smoking. Women and smokers were at higher risk of having neurological disorders with the odds of 1.63 (95% CI: 1.045–2.541) and 1.949 (95% CI: 1.061–3.581), respectively.

Factors affecting cardiovascular system symptoms were sex, smoking, and work section. Women, smokers, working in the sizing and warehouse sections resulted in a higher risk of having cardiovascular disorders with the odds of 1.717 (95% CI: 1.033–2.855), 2.147 (95% CI: 1.029–4.482), 2.516 (1.143–5.538), and 2.826 (95% CI: 1.275–6.264), respectively (Table 4).

## 4 Discussion

This study found that the factors most associated with back and muscular pain was sex. Female workers had more abnormal symptoms than males. This was consistent with the studies by Nagasu et al [16] who revealed that sex was associated with the prevalence of low back pain during 1 month of work (prevalence ratio, *PR* = 1.32; 95% CI, 1.03–1.68) and Tomita et al [17] who studied low back pain in migrant workers who worked in the seafood production industry in Thailand. They found that being female was a risk factor for low back pain (OR = 2.77, CI 95%: 0.79–9.75) and that musculoskeletal disorders were related to working in cold environments [9,18].

We found that age was not associated with low back and muscular pain. Apparently age was a risk factor of back pain, however; the participants in this study were male, mostly around 21–30 years of age, without significantly degenerated spinal bone and intervertebral discs [19]. Moreover, back pain was commonly found in adult workers. Low back pain prevalence was at a peak around the ages of 40–69, and female workers were at higher risk than males [20]. This was not consistent with previous studies which indicated that age was related to low back pain among Thai workers [21] and Western workers [22,23]. Nevertheless follow-up studies in middle age and elderly workers should be conducted.

Sizing, peeling, dissecting, and warehouse workers had more abnormal symptoms than the controls (OR = 5.966, 95% CI: 3.045–11.691; OR = 1.1816, 95% CI: 1.186–2.781; OR = 3.436, 95% CI: 2.097–5.629; OR = 11.962, 95% CI: 6.123–23.445) respectively. Different sections had different cold levels by which the musculoskeletal system could be affected, which was worst in the −10°C environment [2]. Workers in frozen food industries who are repeatedly exposed to cold, humidity, and repetitive work, possibly faced muscle strain [23,24]. Harcombe et al [25] also found that 70% (*n* = 310) of workers had at least one musculoskeletal symptom (OR = 1.35, 95% CI: 1.14–1.6).

Factors affecting symptoms throughout the body were gender, age, and work section in which females had more abnormal symptoms than males (OR = 1.794, 95% CI: 1.2.19–2.641). Elderly workers reported more abnormal symptoms (OR = 0.934, 95% CI: 0.904–0.964). Shivering was normally caused by cold exposure [26]. This study found that workers in extremely low temperatures (−18°C in the warehouse section) experienced more abnormal symptoms than controls (OR = 4.597, 95% CI: 2.002–10.556) regardless of personal protective equipment provided. Physiologically, body temperature regulation caused muscle strain and shivering [4,27].

Cold exposure induced symptoms throughout the body such as discomfort which gradually worsened when the temperature was below −10°C [2], while itching did not occur [4] because when skin temperature was below 20°C this could reduce the symptom by 50% [28].

Factors affecting respiratory symptoms were sex, age, smoking, and work section. Women had more abnormal symptoms than men (OR = 1.888, 95% CI: 1.227–2.905). A previous study indicated a higher prevalence of asthma and bronchitis in female workers. Abnormal symptoms proportionally increased with age [29]. Workers who smoked had more abnormal symptoms than nonsmokers (OR = 1.607, 95% CI: 0.924–2.793) Smoking aggravated respiratory symptoms while working cold environments. Chronic diseases such as musculoskeletal pain, respiratory disease, Raynaud's phenomenon, cardiovascular disease could become worse while working under cold condition [2,5,13,30]. Moreover, smoking was a risk factor of Raynaud's phenomenon [12].

This study indicated that warehouse workers had more abnormal symptoms than controls (OR = 9.731, 95% CI: 3.534–26.797). Cold and dry air inspiration caused acute and chronic symptoms of the upper and lower respiratory tract. Higher morbidity and mortality in the winter [31] was indicated by 160,000 deaths in Michigan with chronic obstructive disease who were at higher risk on colder days [32]. Respiratory disease among employees became worse below −15°C [33], however; differences in sensitivity of each and ventilation were associated with the symptoms [34].

Factors affecting episodic finger symptoms were sex, duration of work, and work section. Female workers had more abnormal symptoms than their counterparts (OR = 1.645, 95% CI: 1.119–2.419). Kaminski et al [35] found that cold sensitivity of the fingers was the chief complaint among can manufacturing workers. Raynaud's phenomenon was mostly found among female workers with gangrenous fingers, toes, nose tip, earlobes, and nipples [36].

Warehouse workers had more abnormal symptoms than controls (OR = 13.514, 95% CI: 5.169–35.327). The temperature in the warehouse was normally lowest at −18°C. Hassi [13], Holmér [4] found that wind speed, humidity, and cold temperature increased the cooling rate of skin and tissues resulting in increasing sensitivity to cold, dermal vasoconstriction especially at the hands, feet, nose, and ears and musculo-skeletal pain in the fingers [2,30]. These abnormal symptoms occurred below −15°C [33].

Factors affecting face and skin symptoms (urticaria) were gender, age, and work section. Those who were female, of older age, and who were working in the warehouse had more abnormal symptoms than controls (OR = 1.932, 95% CI: 0.936–3.987; OR = 3.509, 95% CI: 1.323–9.308; OR = 7.858, 95% CI: 3.171–19.471), respectively. With low enough temperatures, urticaria and reddened and swelled skin could occur [11].

Factors affecting peripheral circulation symptoms were sex and smoking. Women and smokers had more abnormal symptoms (OR = 1.63, 95% CI: 1.045–2.541; OR = 1.949, 95% CI: 1.061–3.581), respectively. Bird et al [37] indicated that cold induced migraine-like headache. The result of this study show that working in the warehouse section was not associated with peripheral circulation symptoms. Abdel-Hamid et al [38] found that those working in the office environment had a higher incidence of headache as a result of poor illumination, bad ventilation, noise, smoking, and dust.

Factors affecting cardiovascular system symptoms were sex, smoking, and work section. Those who were female, smokers, and worked at sizing and in the warehouse had more abnormal symptoms than controls (OR = 1.717, 95% CI: 1.033–2.855; OR = 2.147, 95% CI: 1.029–4.482; OR = 2.516, 95% CI: 1.143–5.538; OR = 2.826, 95% CI: 1.275–6.264), respectively. Exposure to very low temperature would aggravate heart disease. Swoap et al [39] found that ambient air temperatures below 6°C or over 29°C resulted in changes in blood pressure and heart rate of mice. In clinical observation, cold exposure induced sympathetic activities causing a higher risk of hypertension [40]. Moreover, Kawahara et al [41] reported that cold exposure was possibly involved in abnormal heart-indicated parameters.

This study is limited by the relatively short duration of employment. Adverse health effects resulting from working in cold environments usually have a long latency period. Moreover, the abnormal symptoms were self-reported by the individuals. There was no medical evaluation by physicians.

It is suggested that workers' health should be monitored, especially back and muscle pain, respiratory symptoms, darkening of the fingers and toes, and disorders of the heart. As the cofactors of cold-related diseases, those who work in cold environments should avoid smoking and drinking to reduce the risk of cardiovascular disorders.

## Conflicts of interest

All authors declare no conflicts of interest.

## Figures and Tables

**Table 1:** Worker's characteristics

Work sections	Nonexposed	Exposed				
	Office	Sizing	Peeling	Dissecting	Warehouse	Total
	*n* = 255 (%)	*n* = 52 (%)	*n* = 213 (%)	*n* = 167 (%)	*n* = 65 (%)	*N* = 497 (%)
Sex						
Male	56 (22.0)	29 (55.8)	98 (46.0)	83 (49.7)	52 (80)	262 (52.7)
Female	199 (78.0)	23 (44.2)	115 (54.0)	84 (50.3)	13 (20)	235 (47.3)
Age						
Mean (SD) years	31.03 (6.78)	27.77 (6.56)	27.5 (6.60)	27 (6.3)	30.11 (6.99)	27.94 (6.66)
Median (Max, Min) years	30.00 (19–53)	27 (19–48)	26 (15–47)	26 (18–50)	29 (19–50)	27.00 (15–50)
Education						
No education	0 (0.0)	2 (3.8)	23 (10.8)	9 (5.4)	2 (3.1)	36 (7.2)
Elementary (Grade 4/6)	9 (3.6)	28 (53.8)	112 (52.6)	118 (73.3)	11 (16.9)	269 (54.1)
Junior/senior high/diploma	97 (38.1)	74 (42.3)	75 (35.3)	37 (22.2)	41 (63.1)	175 (35.3)
Bachelor degree or higher	149 (58.4)	0 (0.0)	3 (1.4)	3 (1.8)	11 (16.9)	17 (3.4)
Marital status						
Single	149 (58.4)	21 (40.4)	96 (45.1)	70 (41.9)	37 (56.9)	224 (45.1)
Married	91 (35.7)	30 (57.7)	99 (46.5)	91 (54.5)	23 (35.4)	243 (48.9)
Widow/Divorce/Separated	15 (6)	1 (1.9)	18 (8.4)	6 (3.6)	5 (7.7)	30 (6.0)
Smoking history						
Current smoker	20 (7.8)	14 (26.9)	41 (19.2)	36 (21.6)	32 (49.6)	106 (21.3)
Nonsmoker	235 (92.2)	38 (73.1)	172 (80.8)	131 (78.4)	33 (50.8)	371 (74.6)
Mean (SD) (y)	8.20 (4.78)	6.08 (3.32)	9.27 (7.15)	8.30 (6.23)	8.58 (7.51)	8.45 (6.63)
Median (max, min)	8 (2–18)	5 (2–13)	6 (1–29)	6 (2–25)	7 (1–26)	6 (1–29)

**Table 2:** Work history

Factors	Nonexposed	Exposed				
	Office	Sizing	Peeling	Dissecting	Warehouse	Total
	*n* = 255 (%)	*n* = 52 (%)	*n* = 213 (%)	*n* = 167 (%)	*n* = 65 (%)	*N* = 497 (%)
Work duration (y)						
<1	48 (18.8)	19 (36.5)	75 (35.2)	29 (17.4)	9 (13.8)	132 (26.6)
1–5	90 (35.3)	32 (61.5)	138 (64.8)	116 (69.5)	38 (38.5)	324 (65.2)
>5	117 (45.9)	1 (1.9)	0 (0.0)	22 (13.2)	18 (27.7)	41 (8.2)
Mean (SD)	3.47 (4.33)	1.31 (1.32)	1.38 (0.72)	2.65 (2.23)	4.69 (5.54)	2.23 (2.70)
Median (max, min)	1.92 (0.08–24)	1.04 (0.50–10)	1.25 (0.08–4.67)	2 (0.42–9)	2.17 (0.08–22.67)	1.75 (0.08–22.67)
Work hours						
<8	0 (0.0)	0 (0.0)	1 (0.5)	0 (0.0)	1 (1.5)	2 (0.4)
≥8	255 (100)	52 (100)	212 (99.5)	167 (100)	31 (100)	295 (99.6)
Mean (SD)	8.20 (0.60)	8 (0.0)	7.99 (0.14)	8.01 (0.07)	8.11 (0.59)	8.01 (0.237)
Median (max, min)	8 (8–12)	8 (8–8)	8 (6–8)	8 (8–9)	8 (7–12)	8 (6–12)
Work days per week						
5	3 (1.2)	16 (30.8)	41 (19.2)	26 (15.6)	0 (0.0)	83 (16.7)
6	251 (98.4)	36 (69.2)	171 (80.3)	141 (84.4)	63 (96.9)	411 (82.7)
7	1 (0.4)	0 (0.0)	1 (0.51)	0 (0.0)	2 (3.1)	3 (0.6)
Overtime per week (h)						
1–5	4 (1.6)	4 (7.7)	20 (9.4)	4 (2.4)	3 (4.6)	168 (33.8)
6–10	217 (85.1)	48 (92.3)	127 (59.6)	39 (23.4)	52 (80)	43 (8.7)
>10	34 (13.3)	0 (0.0)	66 (31.0)	124 (74.3)	10 (15.4)	2 (0.4)
Mean (SD)	1 (0.0)	3.52 (2.87)	3 (2.58)	3.56 (2.50)	5.21 (3.85)	3.48 (2.72)
Median (max, min)	1 (1–1)	3 (1–12)	2 (1–18)	2 (1–10)	3 (1–14)	2 (1–18)

**Table 3:** Health effects

Symptoms	Nonexposed	Exposed				
	Office	Sizing	Peeling	Dissecting	Warehouse	Total
	*n* = 255 (%)	*n* = 52 (%)	*n* = 213 (%)	*n* = 167 (%)	*n* = 65 (%)	*N* = 497 (%)
Musculoskeletal system (back pain/muscular pain)						
No	208 (81.6)	25 (48.1)	171 (80.3)	102 (61.1)	21 (32.3)	319 (64.2)
Yes	47 (18.4)	27 (51.9)	42 (19.7)	65 (38.9)	44 (67.7)	178 (35.8)
Symptoms throughout the body (discomfort/shivering/itching after cold exposure/entire body cold)						
No	38 (39.6)	28 (57.1)	145 (68.1)	62 (37.6)	12 (18.8)	247 (50.3)
Yes	58 (60.4)	21 (42.9)	68 (31.9)	103 (62.4)	52 (81.3)	244 (49.7)
Respiratory symptoms (asthma/respiratory wheezing/cough/excessive sputum/runny nose)						
No	32 (33.3)	24 (49.0)	125 (58.7)	57 (34.5)	6 (9.4)	212 (43.2)
Yes	64 (66.7)	25 (51.0)	88 (41.3)	108 (65.5)	58 (90.6)	279 (56.8)
Finger symptoms episodic (darkening of fingers/reddening of fingers/finger pain/toe pain/hands and legs sensitive to cold/fingers and toes sensitive to cold)						
No	48 (50.0)	20 (40.8)	150 (70.4)	80 (48.5)	7 (10.9)	257 (52.3)
Yes	48 (50.0)	29 (59.2)	63 (29.6)	85 (51.5)	57 (89.1)	234 (47.7)
Face and skin symptoms (urticaria/face pain)						
No	70 (72.9)	47 (95.9)	199 (93.4)	157 (95.2)	28 (43.8)	431 (87.8)
Yes	26 (27.1)	2 (4.1)	14 (6.6)	8 (4.8)	36 (56.3)	60 (12.2)
Peripheral circulation symptoms (blurry vision/headache/confusion)						
No	47 (49.0)	24 (49.0)	15 (70.9)	135 (81.8)	33 (51.6)	243 (69.9)
Yes	49 (51.0)	25 (51.0)	62 (29.1)	30 (18.2)	31 (48.4)	148 (30.1)
Cardiovascular system (pallor of fingers/chest pain/arrhythmia)						
No	73 (76.0)	30 (61.2)	172 (80.8)	148 (89.7)	42 (65.6)	42 (65.6)
Yes	23 (24.0)	19 (38.8)	41 (19.2)	17 (10.3)	22 (34.4)	22 (34.4)

**Table 4:** Factors affecting abnormal symptoms

	Number	Musculoskeletal system	Symptoms throughout the body	Respiratory symptoms	Finger symptoms episodic	Face and skin	Peripheral circulation symptoms	Cardiovascular system
		aOR (95% CI)	aOR (95% CI)	aOR (95% CI)	aOR (95% CI)	aOR (95% CI)	aOR (95% CI)	aOR (95% CI)
Sex								
Male	318 (42.3)	Ref	Ref	Ref	Ref	Ref	Ref	Ref
Female	434 (57.7)	1.816 (1.186–2.781)	1.794 (1.219–2.641)	1.888 (1.227–2.905)	1.645 (1.119–2.419)	1.932 (0.936–3.987)	1.63 (1.045–2.541)	1.717 (1.033–2.855)
Age (y)	752	–	0.934 (0.904–0.964)	0.96 (0.933–0.988)	0.951 (0.92–0.982)	3.509 (1.323–9.308)	–	–
Alcohol consumption								
Yes	227 (30.2)	0.69 (0.448–1.064)	–	–	–	–	–	–
No	525 (69.8)	Ref						
Smoke								
Yes	117 (15.6)		–	1.607 (0.924–2.793)	–	–	1.949 (1.061–3.581)	2.147 (1.029–4.482)
No	567 (75.4)	Ref		Ref			Ref	Ref
Duration of work (y)	752	–	1.005 (0.99–1.01)	–	1.009 (1.003–1.015)	0.99 (0.99–1.001)	–	–
Work section								
Office	255 (33.9)	Ref	Ref	Ref	Ref	Ref	Ref	Ref
Sizing	52 (6.9)	5.966 (3.045–11.691)	0.638 (0.291–1.395)	0.66 (0.31–1.404)	2.479 (1.13–5.438)	0.11 (0.024–0.51)	1.339 (.643–2.789)	2.516 (1.143–5.538)
Peeling	213 (28.3)	1.433 (0.866–2.371)	0.417 (0.23–0.756)	0.487 (0.277–0.856)	0.742 (0.41–1.349)	0.025 (0.095–0.44)	0.571 (0.332–0.983)	1.026 (0.552–1.907)
Dissecting	167 (22.2)	3.436 (2.097–5.629)	1.336 (0.74–2.415)	1.242 (0.688–2.242)	1.503 (0.843–2.68)	0.144 (0.06–0.346)	0.272 (0.15–0.494)	0.433 (0.212–0.888)
Warehouse	65 (8.6)	11.962 (6.123–23.445)	4.597 (2.002–10.556)	9.731 (3.534–26.797)	13.514 (5.169–35.327)	7.858 (3.171–19.471)	1.596 (0.775–3.287)	2.826 (1.275–6.264)

Factors were removed from the logistic model (*p* > 0.10).
